# Investigation of the effect and availability of ketamine on electroencephalography in cats with temporal lobe epilepsy

**DOI:** 10.3389/fvets.2023.1236275

**Published:** 2023-07-25

**Authors:** Satoshi Mizuno, Rikako Asada, Yoshihiko Yu, Yuji Hamamoto, Daisuke Hasegawa

**Affiliations:** ^1^Laboratory of Veterinary Clinical Neurology, Graduate School of Nippon Veterinary and Life Science University, Musashino, Japan; ^2^Veterinary Medical Teaching Hospital, Nippon Veterinary and Life Science University, Musashino, Japan

**Keywords:** EEG, feline, interictal epileptiform discharge, ketamine, medetomidine, seizure, TLE

## Abstract

In recent years, electroencephalography (EEG) in veterinary medicine has become important not only in the diagnosis of epilepsy, but also in determining the epileptogenic focus. In cats, sedation and immobilization, usually with medetomidine or dexmedetomidine, are necessary to place the electrodes and to obtain stable scalp EEG recordings. In this study, we hypothesized that, for cats with temporal lobe epilepsy (TLE), ketamine, a sedative/anesthetic and N-methyl-D-aspartate (NMDA) antagonist that activates the limbic system and is also used to treat refractory status epilepticus in dogs, would induce sufficient sedation and immobilization for EEG, as well as induce interictal epileptiform discharges (IEDs) that are more pronounced than those induced with medetomidine. We obtained EEG recordings from TLE cats and healthy cats administered either ketamine or medetomidine alone (study 1) or ketamine after medetomidine sedation (study 2). In study 1, the frequency of IEDs showed no statistically significant difference between ketamine and medetomidine in both TLE and healthy cats. Seizures were observed in 75% (9/12) cats of the TLE group with ketamine alone. When ketamine was administered after sedation with medetomidine (study 2), 3/18 cats in the TLE group developed generalized tonic-clonic seizure and 1/18 cats showed subclinical seizure activity. However, no seizures were observed in all healthy cats in both study 1 and study 2. Slow wave activity at 2–4 Hz was observed in many individuals after ketamine administration regardless studies and groups, and quantitative analysis in study 2 showed a trend toward increased delta band activities in both groups. While there was no significant difference in the count of IEDs between medetomidine and ketamine, ketamine caused seizures in cats with TLE similar to their habitual seizure type and with a higher seizure frequency. Our results suggest that ketamine may activate epileptiform discharges during EEG recordings. However, caution should be used for cats with TLE.

## 1. Introduction

Ketamine (KET) is a noncompetitive N-Methyl-D-aspartate (NMDA) receptor antagonist that was developed in the 1960s. Corssen et al. (1) reported that the corticothalamic system is suppressed while the limbic system is activated in cats using KET, thus, KET is referred to as a “dissociative anesthetic.” In veterinary medicine, KET is generally used for restraint, sedation, and analgesia. In addition to these applications, KET has been recently used to control refractory (GABAergic drugs-resistant) status epilepticus (RSE) in dogs (2). However, studies on the effects of KET on epilepsy are inconsistent, with reports stating that KET has antiseizure effects (3, 4), while other reports state that KET induces seizure activity (5).

Electroencephalography (EEG) is one of the most important diagnostic tools in epilepsy and has also attracted attention for detecting the epileptogenic zone for both seizure classification and epilepsy surgery (6, 7). However, in the veterinary field, especially in cats, EEG usage is not prevalent. As one factor of this, EEG in cats with high-density coats and hard scalps requires strict sedation and immobilization for electrode placement and stable recording. Some studies have reported the use of sedatives, typically medetomidine (MED) or the enantiomer of MED, dexmedetomidine, to perform scalp EEG in dogs and cats (8–10). MED and dexmedetomidine produce sedative and analgesic effects enough for EEG recording and can be rapidly antagonized by the administration of atipamezole (11, 12). Therefore, MED or dexmedetomidine is preferred for EEG recording in animals. In a canine EEG study with MED, dogs with severe seizures had a predominantly higher incidence of interictal epileptiform discharges (IEDs) than control dogs (13). However, there are cases in which IED cannot be detected in actual EEG measurements, even if the patient has epilepsy, and repeated EEG testing is recommended depending on the case (14). However, because of the burdens associated with EEG in animals, including the administration of sedatives, an agent that is safe and does not interfere with IED detection would be ideal.

In previous studies, we reported familial spontaneous epileptic cats (FSECs) with suspected genetic epilepsy. FSECs have spontaneous focal limbic seizures with or without evolving to generalized tonic-clonic seizures (GTCS), and, therefore, are classified as temporal lobe epilepsy (TLE) (15–21). The epileptogenic zone of FSECs is presumed to exist in the hippocampus or amygdala, which are parts of the limbic system, or both based on the previous evaluations of symptomatogenic, irritative, seizure-onset, structurally abnormal, and functional deficit zones (22).

Although feline EEG using KET was examined in the past (4, 5, 23–26), to our knowledge, there are no reports of such studies conducted in cats with spontaneous TLE. Therefore, we hypothesized that the use of KET, which stimulates the limbic system and inhibits the cortex, in EEG in cats with TLE would increase the number of IEDs. To verify this hypothesis, we conducted scalp EEG in healthy and TLE cats after the sole administration of KET or MED (study 1). Then, according to the results from study 1, we evaluated the effect of KET after sedation with MED on scalp EEG in cats with TLE (study 2).

## 2. Materials and methods

### 2.1. Animals

This study, including the care and maintenance of the FSECs colony, healthy, and epileptic cats genetically unrelated to FSECs, was approved by the Animal Care and Use Committee of Nippon Veterinary and Life Science University (accession Nos. 2020K-3, 2021K-2, 2022K-2; the principal investigator is DH).

The study included 24 cats, divided into two groups: the TLE group and the control group. The TLE group included 17 FSECs (Nos. 1–17) and one cat with TLE not related to FSEC strain (No. 18). FSECs show the typical seizure type of feline TLE such as orofacial automatism, salivation, mydriasis, head-turning, and sometimes evolve to GTCS (27). Both physical and neurological examinations were normal. Cat No. 18 had an initial seizure at the age of 50 months and presented focal limbic seizures with or without evolving to GTCS similar to FSECs. Physical and neurologic examinations, complete blood count, serum chemistry, urinalysis, and magnetic resonance imaging showed no abnormalities, and previous EEG revealed IEDs (spikes) in the left anterior temporal region. These cats were observed with seizures for more than 1 year using a video monitoring system. In the TLE group, seizures were observed in cats No. 1 (12 seizures/year), No. 2 (19 seizures/year), No. 4 (1 seizure/year), and No. 18 (14 seizures/year), while other cats had no seizures in the year before inclusion in this study. All cats have not been treated with daily antiepileptic seizure medication; however, they received temporary treatment for severe cluster seizures and/or status epilepticus as appropriate. The TLE group consisted of 12 males and 6 females (3 of which were neutered), and had a median age and body weight of 127 months (range, 94–157) and 3.6 kg (range, 2.5–7.2), respectively. The control group included 6 healthy cats (Nos. 19–24; 2 males and 4 females) without any documented seizures. The control group had a median age and body weight of 94 months (range, 77–107) and 3.6 kg (range, 3.1–6.4), respectively. Signalment and seizure frequency for each individual at the time of study inclusion are summarized in Supplementary Table1.

### 2.2. Study 1: EEG by sole administration of ketamine or medetomidine

#### 2.2.1. Sedation protocol

Twelve cats (Nos. 1–5 and 18 from the TLE group, and Nos. 19–24 from the control group) were subjected to EEG with the sole administration of either MED or KET on a separate day for each individual. All cats were restricted in feeding and drinking for 12 h before EEG recording. EEG was performed once with MED (Domitor^®^, Zenoaq, Japan) alone. EEG was also performed once with KET (Ketalar^®^, Daiichi-Sankyo, Japan) alone, but performed again (i.e., twice) on another day if epileptic seizures occurred or if sufficient EEG recordings could not be obtained, to confirm reproducibility. Each test was performed at least 1 week apart. MED (median dose, 40 μg/kg; range, 30–50) or KET (median dose, 7.5 mg/kg; range, 5.0–13.3) was administered intramuscularly according to the sedation status of each individual. All cats sedated with MED received an intramuscular administration of the MED antagonist atipamezole (Antisedan^®^, Zenoaq, Japan) after the EEG recording.

#### 2.2.2. EEG recordings

**Figure 1:**
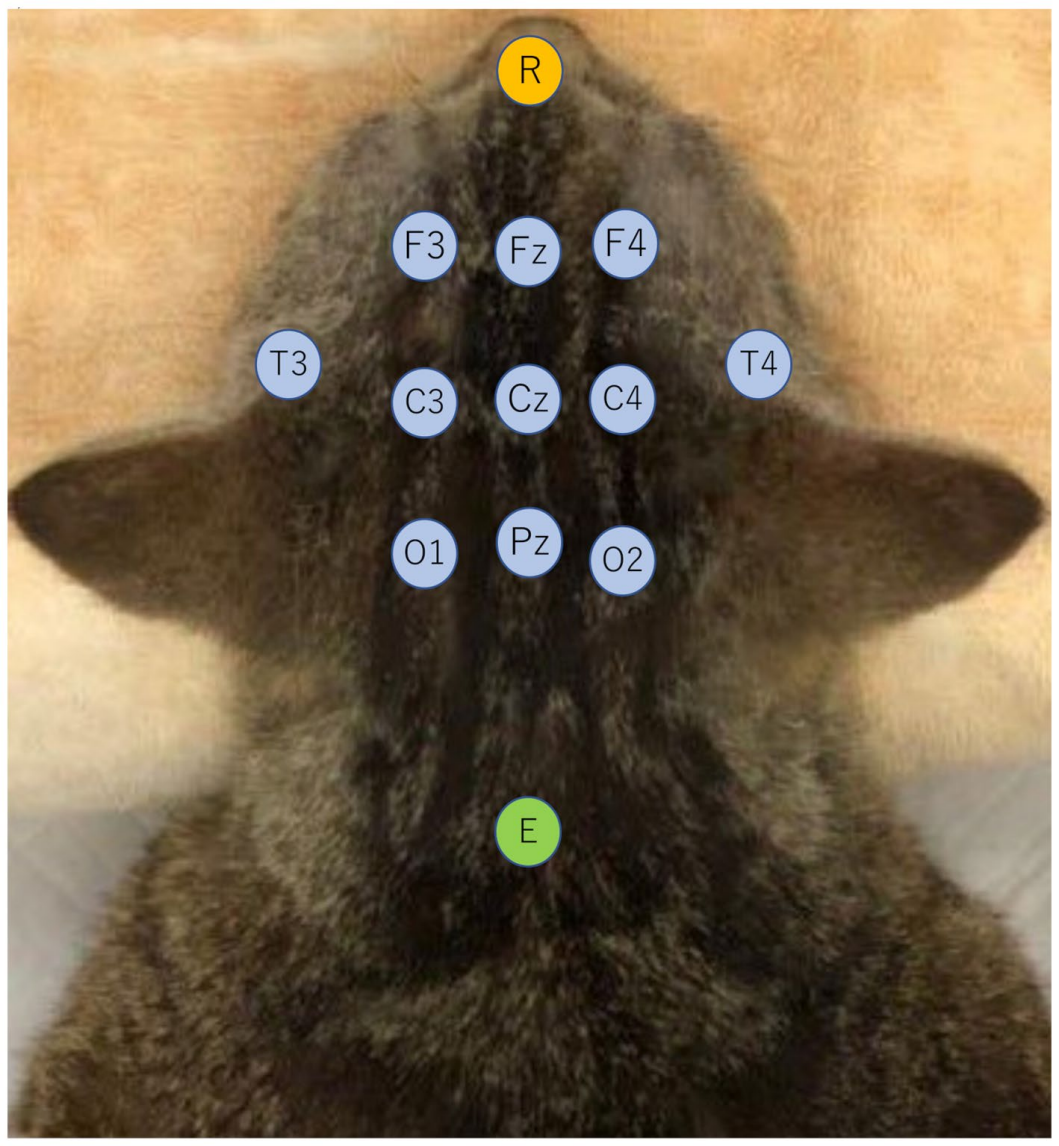
Electrode placement viewed from the dorsal aspect of a cat. Recording electrodes are bilateral frontal (F3/F4), central (C3/C4), temporal (T3/T4), occipital (O1/O2), and 3 longitudinal midline electrodes (Fz, Cz, Pz) indicated in light blue. The reference electrode (R) was placed on the dorsal surface of the nasal tip, indicated in orange, and the ground electrode (E) was placed at the level of the axial spinous process, indicated in green.

EEG recordings were conducted while the cats were under adequate sedation. After the cats were placed in the prone position, recording needle electrodes were placed subcutaneously on the bilateral frontal (F3/F4), central (C3/C4), temporal (T3/T4), and occipital (O1/O2) regions, as well as three midlines (Fz, Cz, Pz), and the reference and the ground electrodes were placed at the apex of the nose and the neck, respectively (20) (Figure 1). EEGs were measured with a digital EEG unit (Neurofax^®^EEG-1200, Nihon Kohden, Japan) under following parameters: sampling frequency, 1,000 Hz; sensitivity, 5–10 μV/mm; time constant, 0.1–0.3 s; Hi-cut filter, 60 Hz; and total recording time, 20–30 min. Electrocardiogram was monitored simultaneously using bipolar electrocardiogram leads in the same EEG unit.

#### 2.2.3. Visual analysis of EEG

In the present study, we define and use “incidence” as “the rate of occurrence” and “frequency” as “Hz.”

The recorded EEG montage was set to reference derivation and bipolar derivation, including average reference derivation. Stable recorded periods were randomly selected and evaluated for a total of 5–10 min to assess the incidence of IED per minute. One EEG reading-trained veterinarian (SM) counted IEDs. If the stable recorded periods were less than 5 min, they were excluded from the analysis. If a seizure occurred before sufficient EEG could be recorded, the evaluation of IEDs in the EEG measurements under KET sedation was performed after the seizure had ended. IEDs including spikes, polyspikes, spike-and-waves, and sharp-waves were counted and the region(s) with the highest incidence of IEDs was identified. When an IED was completely synchronized in all derivations, it was counted as one for all regions. The incidence of IEDs (IEDs/min) in each group was calculated, and the presence or absence of seizures after drug administration and their details were recorded. If seizures were observed after the administration of KET, EEG measurements were performed again on another day, and the average of the two measurements was analyzed as the IEDs for that individual. The incidence of IEDs in each group was analyzed by Wilcoxon signed rank test. Statistical analyses were performed using R version 4.1.0 (The R Foundation for Statistical Computing, Vienna, Austria). The significant difference in statistical analysis was defined as *p* < 0.05.

### 2.3. Study 2: EEG with ketamine administration after medetomidine sedation

#### 2.3.1. Sedation protocol and EEG recording

Based on the results of study 1, we had planned to investigate the potential utility of KET as an EEG activator, i.e., increasing IED or inducing seizure activity, on scalp EEG under MED sedation. Twenty-four cats (Nos. 1–18 from the TLE group and No. 19–24 from the control group) were included. All cats were restricted in feeding and drinking for 12 h before the administration of MED. EEG recording was started after the cat was sedated by intramuscular MED and EEG was recorded for ≥10 min (MED), then KET was administered intravenously and EEG was recorded for another ≥10 min (MED-KET). The median dose of MED until each individual was sufficiently sedated to perform EEG was 30 μg/kg (range, 30–50). The dose of KET was 1.0 mg/kg, however, for cats (Nos. 2 and 18) that developed status epilepticus in study 1, the dose was decreased to 0.25 mg/kg. And for cat No. 3, which was not adequately sedated after 1.0 mg/kg KET administration, an additional dose of 0.5 mg/kg was given. Finally, the median dose of intravenous KET was 1.0 mg/kg (range, 0.25–1.5). After 20–30 min of recording of EEG following KET administration, an adequate dose of atipamezole was administered intramuscularly. The recording conditions of EEG were the same as described in study 1.

#### 2.3.2. Visual analysis of EEG

Montages of the recorded EEG were constructed in the same manner as in study 1. Stable recordings obtained in MED and MED-KET periods were randomly selected and evaluated for each 5–10 min to assess the incidence of IEDs per minute. If the total time of evaluable recordings was <5 min after the administration of KET, the recordings were excluded from the analysis. For counting IEDs and identifying the highest incidence regions, the same methods were used as described in study 1. The incidence of IEDs between MED and MED-KET periods in each group was analyzed by Wilcoxon signed rank test. The significant difference in statistical analysis was defined as *p* < 0.05.

#### 2.3.3. Quantitative analysis of EEG

Quantitative EEG analysis was set to average reference derivation. In both MED and MED-KET periods, a 2 s artifact-free period was visually selected (3 or 4 parts) during each recording period, and background activity was analyzed using a fast Fourier transform (FFT). Spectral bands were 0.5–4.0 Hz for delta band, 4.1–8.0 Hz for theta band, 8.1–13.0 Hz for alpha band, and 13.1–30.0 Hz for beta band. The relative power of the spectral bands was calculated for all leads and averaged. We analyzed each lead in each individual using Wilcoxon signed rank test. The significant difference in statistical analysis was defined as *p* < 0.05.

## 3. Results

### 3.1. Study 1: EEG by sole administration of ketamine or medetomidine

EEG recordings were obtained from all 12 cats administered MED only and were performed only once in each cat. On the other hand, EEG recordings of cats with KET administration alone were obtained for 10/12 cats. Two cats were excluded from the EEG analysis: No. 18 from the TLE group experienced a seizure induced by KET administration, which lasted longer than 30 min (i.e., developed to status epilepticus): and KET administration could not induce sufficient sedation in No. 22 from the control group.

During the EEG recording with MED alone, no TLE or healthy cats showed seizures. However, when KET alone was administered, all cats in the TLE group (6/6) showed clinical seizures, thus, EEG with KET alone was performed twice in all TLE cats (a total of 12 recordings). Seizures were observed in TLE cats in 9 of the 12 recordings (75%), 6 of which progressed to GTCS. In some TLE cats (3/6 cats, 5/12 recordings), seizures did not end within 5 min spontaneously, so antiseizure medications (diazepam, midazolam, levetiracetam) were used to stop the seizure. The seizure signs observed after KET administration overlapped in part with those observed in spontaneous habitual seizures in 5/6 cats (Table 1). On the other hand, there was no cat that showed any seizures in the control group.

**Table 1:** Seizure signs observed during intramuscular ketamine administration and spontaneous seizures in temporal lobe epilepsy group.

Cat (No.)	Ketamine-induced	Spontaneous (habitual seizure)
1	Type 1^a^: orofacial automatism, urination, hypersalivation, mydriasis, GTCS	Looking around, mydriasis, orofacial automatism, head turning, GTCS
	Type 2^a^: orofacial automatism, GTCS	
2	Type 1^a^: looking around, forward leaning posture, mydriasis, GTCS	Looking around, nodding, facial twitching, head turning, GTCS
	Type 2^a^: looking around, orofacial automatism, GTCS	
3	Type 1^a^: mydriasis, orofacial automatism	Orofacial automatism, GTCS
4	Type 1: facial twitching	Mydriasis, urination, head turning, GTCS
5	Type 1^a^: myoclonic seizures	Facial twitching, orofacial automatism, myoclonic seizures, GTCS
18	Type 1^a^: orofacial automatism, GTCS	Looking around, mydriasis, hypersalivation, orofacial automatism, GTCS

GTCS, generalized tonic-clonic seizures.

a Ketamine-induced seizures had a similar seizure sign to the spontaneous seizures.

**Figure 2:**
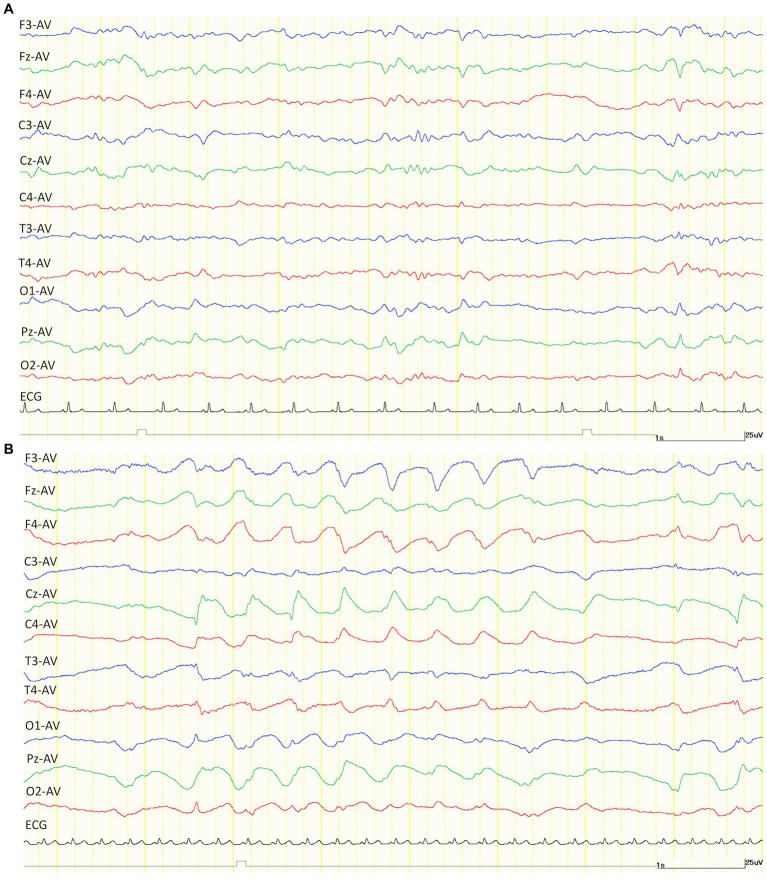
Examples of EEG trace after intramuscular administration of medetomidine (30 μg/kg) alone **(A)** and ketamine (7.5 mg/kg) alone **(B)**. Compared to medetomidine **(A)**, the EEG after administration of ketamine showed a generalized slow wave of about 2 Hz. Both EEG montages were set to average (AV) reference derivation. ECG, electrocardiogram.

After the administration of KET, generalized slow waves of 2–4 Hz were observed intermittently in 4/5 cats of the TLE groups that were available for analysis (Figure 2). Since the number of slow waves tended to decrease with time, the maximum number of slow waves observed in each animal was determined by measuring 1 min from the time the slow waves were first observed. The average incidence of slow waves in the TLE cats was 26.3 cycles/min. Those intermittent slow waves lasted 20–40 min after KET administration. Similarly, intermittent slow waves of 2–5 Hz were observed in 2/5 cats of the control group. Slow waves were measured in the same way as in the TLE group, the average incidence of slow waves in the control group was 26.5 cycles/min observed over 20 min after KET administration.

**Figure 3:**
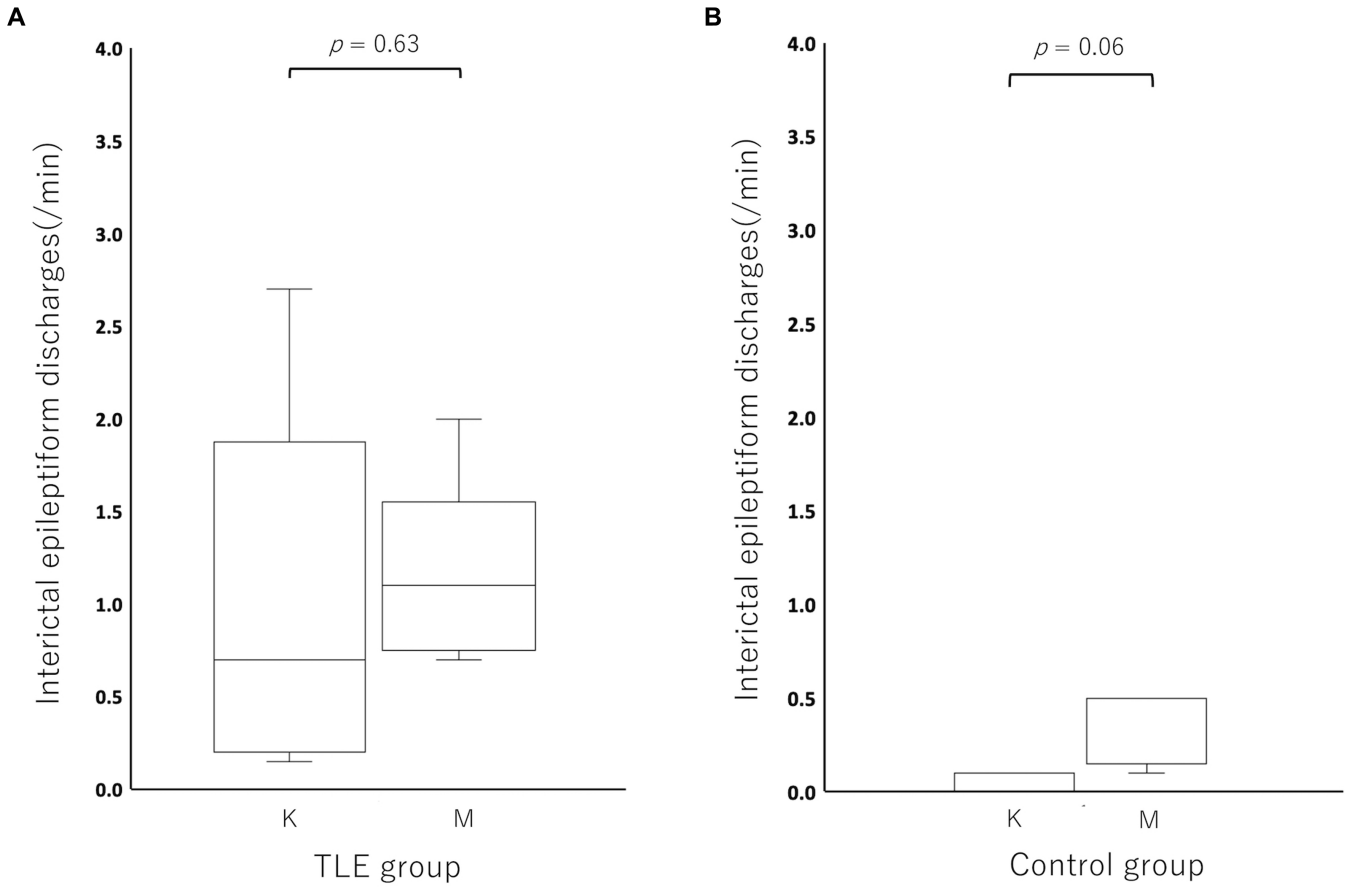
Boxplots for comparing the incidence of interictal epileptiform discharges between ketamine and medetomidine in the temporal lobe epilepsy (TLE) group **(A)** and control group **(B)**. There was no significant difference in IEDs between ketamine and medetomidine in the temporal lobe epilepsy group (*p* = 0.63) or in the control group (*p* = 0.06). K, ketamine; M, medetomidine.

Compared to the incidence of IED under MED in each individual, the incidence of IED under KET in the TLE group was activated (increased) in 2/5 cats and depressed (decreased) in 3/5 cats (KET: median, 0.70; range, 0.15–2.70; interquartile range (IQR), 0.25–1.05 vs. MED: median, 1.10; range, 0.70–2.00; IQR, 0.80–1.10). The incidence of IED under KET was depressed in all cats of the control group (5/5) (KET: median, 0.00; range, 0.00–0.10; IQR, 0.00–0.10 vs. MED: median, 0.50; range, 0.10–0.50; IQR, 0.20–0.50). There was no significant difference in the incidence of IED between KET and MED in both TLE (*p* = 0.63) and control groups (*p* = 0.06) (Figure 3). The incidence and highest incidence region of IEDs are summarized in Table 2. In the TLE group, the regions with the highest incidence of IEDs were compared between KET and MED, and agreement was found in 3 of 5 cases.

**Table 2:** The incidence and the highest incidence region of interictal epileptiform discharges under ketamine or medetomidine.

Cat (No.)	Highest incidence region of IEDs		Incidence of IEDs (/min)	
	Ketamine	Medetomidine	Ketamine	Medetomidine
*Temporal lobe epilepsy group*				
1	Left temporal	Left temporal	2.7	2.0
2	Left temporal	Left temporal	0.7	1.1
3	Left occipital	Right central	0.25	1.1
4	Left temporal	Right temporal	0.15	0.8
5	Left temporal	Left temporal	1.05	0.7
18	NE	Left temporal	NE	0.8
*Control group*				
19	Left central	Median central, Left central	0.1	0.2
20	Right central	Left central	0.1	0.5
21	—	Left temporal	0.0	0.1
22	NE	Left frontal	NE	1.0
23	—	Left central	0.0	0.5
24	—	Left central	0.0	0.5

NE, not evaluable; IEDs, interictal epileptiform discharges.

### 3.2. Study 2: EEG with ketamine administration after medetomidine sedation

EEG records were available for 23/24 cats, with one cat (No. 18 from the TLE group) excluded from the EEG analysis after having a seizure lasting more than 30 min (status epilepticus) after the administration of KET.

**Figure 4:**
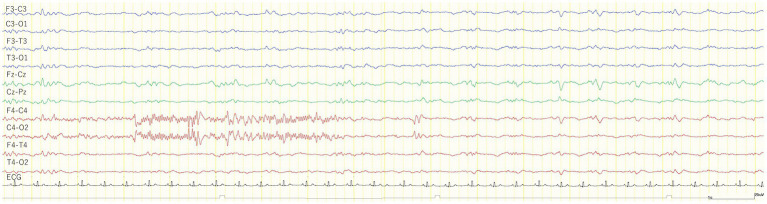
EEG trace of a subclinical seizure observed 7 min after the intravenous administration of 1 mg/kg ketamine during medetomidine sedation (cat No. 7). The cat was immobilized and no body movements were observed before and after a subclinical seizure. The recorded EEG montage was set to bipolar derivation. ECG, electrocardiogram.

All 24 cats had no seizures after MED administration, whereas three cats (Nos. 1, 2, and 18) in the TLE group had GTCS after KET administration. In addition, a subclinical focal seizure activity was observed in another cat (No. 7) of the TLE group (Figure 4). The site of this subclinical seizure occurrence coincided with the site’s highest incidence of IEDs under MED and KET. Of 23 cats that were available for analysis after KET administration, 18 cats had slow waves (12/16 from the TLE group, 6/6 from the control group). As in study 1, intermittent generalized slow waves of 1–4 Hz were observed within 1 min after KET administration. Slow waves tended to decrease with time and were measured in the same way as in study 1. The average incidence of slow waves in the TLE group was 43.8 cycles/min, and the average duration was 9.1 min. The average incidence of slow waves and the average duration in the control group were 43.3 cycles/min and 13.2 min, respectively. Changes in EEG findings observed after KET administration are summarized in Table 3.

**Table 3:** Changes in EEG activity after ketamine administration during medetomidine sedation.

Cat (No.)	EEG findings after ketamine administration	Incidence of slow wave (cycles/min)	Duration of slow wave (min)
*Temporal lobe epilepsy group*			
1	1–2 Hz GSW	18	8
2	No clear change was observed	—	—
3	No clear change was observed	—	—
4	2–3 Hz GSW	72	11
5	2 Hz GSW	23	5
6	2–4 Hz GSW	10	4
7	3 Hz GSW	58	14
8	No clear change was observed	—	—
9	No clear change was observed	—	—
10	2 Hz GSW	32	3
11	1–2 Hz GSW	60	6
12	2 Hz GSW	63	13
13	1 Hz GSW	45	20
14	2–3 Hz GSW	46	10
15	2 Hz GSW	49	8
16	2 Hz GSW	49	7
17	No clear change was observed	—	—
18	NE	—	—
*Control group*			
19	2 Hz GSW	43	20
20	1–2 Hz GSW	59	12
21	1–2 Hz GSW	47	13
22	2–3 Hz GSW	63	15
23	2 Hz GSW	21	13
24	2–3 Hz GSW	27	6

Incidence of slow wave: the count of waves during the first 1 min after it was observed. Duration of slow wave: the duration time from observation of slow waves to disappearance. NE, not evaluable; GSW, generalized slow wave.

**Figure 5:**
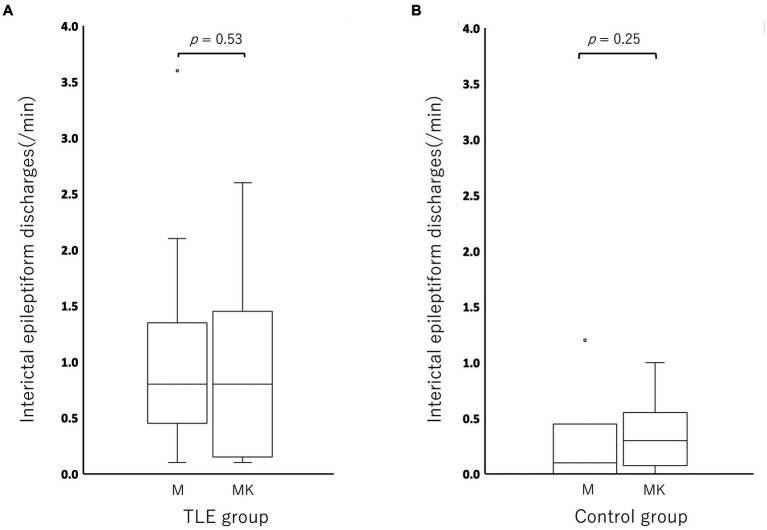
Boxplots for comparing the incidence of interictal epileptiform discharge (IED) between medetomidine and ketamine following medetomidine in the temporal lobe epilepsy (TLE) group **(A)** and control group **(B)**. Circles at the top of the box represent outliers. There was no significant difference between medetomidine alone and ketamine following medetomidine in the TLE group (*p* = 0.52), or in the control group (*p* = 0.25). M, medetomidine; MK, ketamine administration following medetomidine sedation.

Compared to the MED period in each individual, the incidence of IEDs of the MED-KET period in the TLE group was activated in 5/17 cats, depressed in 9/17 cats, and unchanged in 3/17 cats (MED: median, 0.80; range, 0.10–3.60; IQR, 0.50–1.10 vs. MED-KET: median, 0.80; range, 0.10–2.60; IQR, 0.20–1.30). In the control group, the incidence of IED was activated in 4/6 cats, depressed in 1/6 cats, and unchanged in 1/6 cats (MED: median, 0.10; range, 0.00–1.20; IQR, 0.03–0.18 vs. MED-KET: median, 0.30; range, 0.00–1.00; IQR, 0.13–0.40). There was no significant difference in the incidence of IED between MED and MED-KET regardless group (TLE group, *p* = 0.53; control group, *p* = 0.25) (Figure 5). The incidence and highest incidence region of IEDs are summarized in Table 4. In the TLE group, agreement of the regions of the highest incidence of IEDs between MED and MED-KET was observed in 10 of 17 cases.

**Table 4:** The incidence and the highest incidence region of interictal epileptiform discharges under medetomidine alone and ketamine following medetomidine.

Cat (No.)	Highest incidence region of IEDs		Incidence of IEDs (/min)	
	Medetomidine	Medetomidine-ketamine	Medetomidine	Medetomidine-ketamine
*Temporal lobe epilepsy group*				
1	Left temporal	Left temporal	1.8	2.6
2	Left temporal	Right temporal	0.7	0.2
3	Right central	Left temporal	0.8	0.1
4	Right temporal	Right temporal	1.0	1.7
5	Left central	Left central	1.1	0.7
6	Left occipital	Left temporal	1.6	1.6
7	Median frontal	Right central	0.6	1.3
8	Right temporal	Right temporal	1.1	1.0
9	Right temporal	Right temporal	0.5	0.8
10	Left temporal	Left temporal	3.6	1.2
11	Left central	Right temporal	0.4	0.1
12	Right temporal	Right temporal	0.5	0.6
13	Left central	Left central	2.1	1.9
14	Left central	Left temporal	0.3	0.3
15	Right central	Right central	0.1	0.1
16	Right temporal	Right temporal	0.3	0.1
17	Left temporal	Left temporal	1.1	0.9
18	Left temporal	NE	0.9	NE
*Control group*				
19	Left temporal	Left temporal	0.1	0.4
20	—	Right temporal	0.0	0.1
21	—	—	0.0	0.0
22	Left central	Left central	1.2	1.0
23	Left temporal	Left temporal	0.2	0.4
24	Left frontal	Left temporal, Left frontal	0.1	0.2

NE, not evaluable; IEDs, interictal epileptiform discharges.

**Figure 6:**
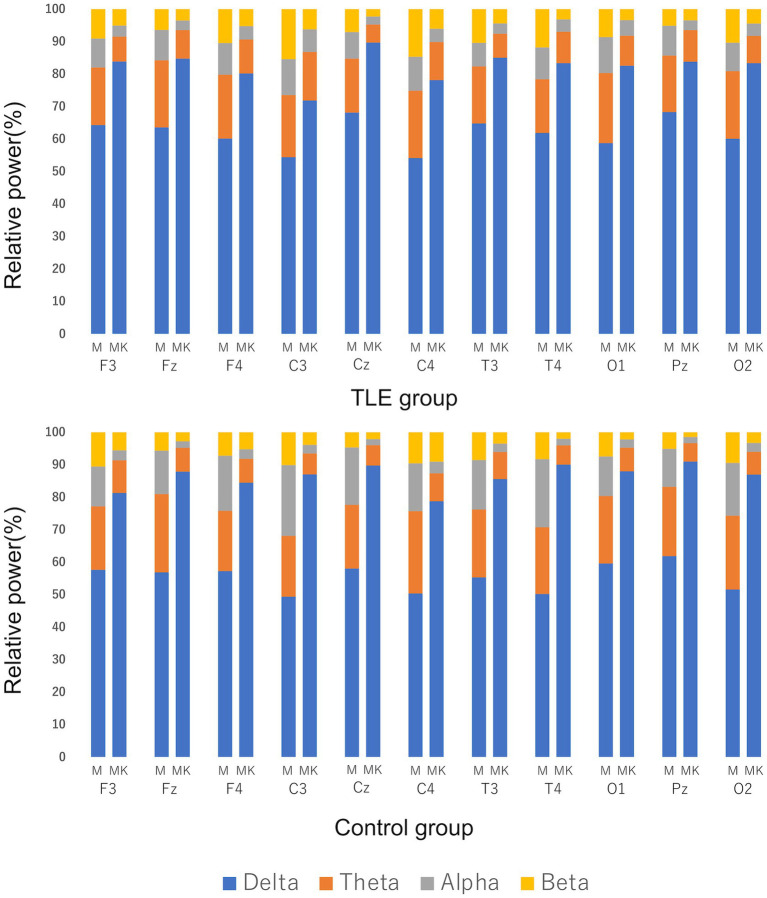
Comparison of median relative power using EEG recordings of medetomidine alone (M) and ketamine following medetomidine (MK). Relative power was defined as delta band (0.5–4.0 Hz), theta band (4.1–8.0 Hz), alpha band (8.1–13.0 Hz), and beta band (13.1–30.0 Hz). Vertical lines indicate the percentage of the relative power during M and MK at each site. There was a clear increase in the percentage of delta bands in both the temporal lobe epilepsy (TLE) group and the control group.

For the quantitative analysis, 17 cats from the TLE group and 6 cats from the control group were included. Cat No. 18 was excluded from the analysis because sufficient EEG activity could not be recorded due to seizure activity. In both groups, there was a trend toward increased delta activity after KET administration when median relative power was compared (Figure 6). Significant differences between MED and MED-KET were observed in all sites, except for the theta band of C3 and the beta of F3, Fz, F4, and C4 in the TLE group, and the theta of F3 and beta of F3 and C4 in the control group (Supplementary Table 2).

## 4. Discussion

In this study, we investigated the effects of intramuscular KET alone or intravenous KET under MED sedation on scalp EEG in healthy cats and cats with TLE. There were no significant differences in the incidence of IEDs between MED and KET nor between MED and MED-KET. In a previous report, KET suppressed focal seizures in 6 of 16 cats with epileptogenic focus produced by penicillin, and it did not affect IEDs in 10 cats (4). On the other hand, studies using KET in baboons have reported generalized IED activation in the scalp EEG (28). In the present study, the incidence of IEDs tended to be depressed by both KET alone and MED-KET in both control and TLE groups. Taking into account the previous study that KET suppressed the corticothalamic system and activated the limbic system (1), the trend in decreasing the incidence of IEDs in the present study may suggest that KET has an inhibitory effect on the corticothalamic system in cats. Although there were no statistically significant differences to support these trends in the present analysis, one factor may be the exclusion of individuals who did not have sufficient EEG records, especially with respect to the administration of KET alone.

In the TLE group, clinical seizures were observed in all cats after the administration of KET alone. The KET-induced seizure in 5 of 6 TLE cats was similar to their habitual seizures, i.e., limbic seizure to generalization (see Table 1 for details). In addition, a subclinical seizure was observed in one TLE cat (No. 7) after the administration of KET following MED sedation, the onset of which coincided with the regions with the highest incidence of IEDs. Although the mechanism by which KET induces seizures has not been elucidated, these facts suggest that KET activated the limbic system, which is the epileptogenic zone of TLE cats in this study. In addition, the three cats (Nos. 1, 2, and 18) that had clinical seizures induced by KET in both studies had a higher seizure frequency (12, 19, and 14 per year, respectively) than the others. Thus, whether the epileptogenic zone is activated with KET administration may depend on seizure frequency.

In the adult human, the detection rate of epileptic discharges is reported to be approximately 50% on the initial examination and increases to approximately 80%–90% with repeated testing (29). However, unlike humans, EEG in cats always requires immobilization using sedatives or anesthetics, and few EEGs are repeated. In addition, a recently developed ambulatory EEG system for dogs and humans may not be adopted for cats due to the nature of felines and the size and weight of the equipment. In this situation, capturing clinical seizure onset during a single scalp EEG is often difficult, depending on the individual’s seizure frequency. A previous report suggested that low doses of KET may lower seizure thresholds within minutes of administration in people with a predisposition to focal epilepsy (30). Considering that we were able to capture seizures after KET administration compared to MED alone in the present study, KET administration may be useful for determining the epileptogenic zone.

In both studies of KET administration alone (study 1) and KET administration after MED (study 2), the generalized slow wave was observed in both TLE and control groups. Quantitative analysis showed that the delta band on background activity in both TLE and control groups was significantly increased, while other bands were significantly decreased in most areas. In a study with cats, electrocorticogram changes during KET administration under general anesthesia with ether were reported to occur in a dose-dependent manner, with doses below 5 mg/kg causing generalized beta activity and doses above 5 mg/kg causing alternating bursts of polyspike-slow waves and high-potential synchronous beta waves (4). In humans, low doses of KET were reported to cause the EEG to show fast oscillations in the high beta and low gamma, and that the slow oscillations exhibited by KET were more irregular than the slow oscillations caused by propofol and dexmedetomidine (31). That report also suggested that the beta and gamma oscillations were caused by KET’s preference for NMDA receptors on inhibitory interneurons, which, in turn, increased cerebral metabolic rate, cerebral blood flow, and hallucinations (31). However, in the present study, we did not observe beta oscillations, and, in the quantitative analysis of study 2, beta oscillations were clearly reduced and a visually recognizable generalized slowing (2–5 Hz) was observed in many cats after KET administration. The slow waves observed in study 1 may be due to cortical inhibition by KET, and the lack of fast oscillations as reported in the past (under anesthesia) may be due to insufficient immobilization by KET alone, which may have masked these fast oscillations. The reason why beta oscillations were reduced in study 2 and slow waves were observed in more individuals than in study 1 (6/10 vs. 18/22) may be due to cortical inhibition by KET and the pre-administration of MED. High-voltage and low-frequency activity expressed as delta and theta rhythms have been reported as MED-induced background activity in cats (9). In addition, it has been reported that intraperitoneal administration of MED and KET in rats increases low-frequency bands with only minor changes in the high-frequency bands (32). The KET-induced higher frequency band activity has been reported to be associated with increased cerebral metabolic rate and cerebral blood flow by inhibiting NMDA receptors, as described above, while alpha-2 agonist (MED) has been reported to decrease cerebral blood flow (33). Therefore, fast oscillations in the high beta and low gamma may have been suppressed in the present study.

In previous studies in normal cats or feline experimental models receiving KET, KET was administered under anesthesia, and changes in EEG were investigated (4, 5, 23–26). One of these reports evaluated the effect of KET on IEDs in the penicillin-induced seizure model of cats; however, quantitative evaluation has not been conducted (4). Another study reported that 5 of 22 (23%) healthy cats administered an anesthetic dose of KET (20 mg/kg IM) showed limbic seizures evolving to GTCS (23). However, to the best of our knowledge, there is no report of KET administration in cats with naturally occurring TLE so far. In the present study, we used cats with naturally occurring TLE to assess background activity, the presence of seizure activity, and the quantitative of IEDs under KET-induced sedation. We consider that the results of the present study provide important consideration for the use of KET in cats in the veterinary clinical setting.

We recognize there are several limitations to the present study. First, in the comparison of IEDs between KET and MED, most of the IED counts for KET in the TLE group were conducted in post-ictal EEG measurements because of a seizure induced immediately after KET administration. It is known that postictal generalized EEG suppression (PGES) occurs immediately after an epileptic seizure (34). PGES are generally observed more frequently in convulsive seizures than in focal seizures, but reports on the frequency of occurrence and duration are very inconsistent (35). Therefore, PGES may have occurred after KET-induced seizures and reduced the number of IED in the present study. Second, we considered the dose variation of agents to obtain sufficient sedation for EEG recording as a possible reason for the variability in the results of this study. Although KET can produce dose-dependent EEG changes as mentioned above (4), the dose of KET alone in this study was at the upper limit of a typical sedation dose because it was intended to sedate sufficiently for measuring EEG and to avoid respiratory depression. Therefore, the large dose variability among individuals may have particularly affected the analysis. Because of individual differences in sedative effects, the administration of KET alone may result in a time lag or inadequate sedation before an EEG measurement can be performed. If the time lag between the administration and EEG measurement was significant, a subclinical seizure may have been missed. To confirm the acute effects of KET immediately after administration, as in this study, simultaneous administration of a sedative such as MED may be necessary. Alternatively, EEG under intubation should be considered, given the possibility of respiratory depression. When an anesthetic dose of KET was administered, we expected that the reported bursts of polyspike-slow waves (i.e., elevated beta and delta bands) would be observed (4). However, in recent years, it suggested that the administration of KET under appropriate respiratory control might suppress EEG activity (36). Therefore, if appropriate respiratory management is performed after the administration of KET, a remarkable alteration in the power spectrum may be observed. Further investigation of the dose-depend effect of KET on EEG is necessary. Third, the fact that many cats included in the present study were older, and that there was a wide variation in seizure frequency prior to inclusion may have contributed to the variability in the results. An EEG study using two groups of cats divided according to age under sedation with MED reported that older cats had significantly higher relative power in the theta, alpha, and beta bands and lower in the delta band than younger cats (9). Thus, in our MED-KET study, the significant increase in the delta band might have been more pronounced in our older cats than in a young cat population.

In the present study, MED produced more stable sedation than KET alone in cats with TLE, and EEG measurements were feasible. Furthermore, KET is easy to induce seizures in cats with TLE. Therefore single-use KET as a sedative for EEG is not recommended from the results of this study. However, we would suggest that KET may be useful to activate seizure activities on EEG under MED sedation. This will be a critical tool to obtain ictal EEG for detecting the epileptogenic zone (irritative or seizure-onset zones). Although the combination of MED and KET has long been used as an anesthetic induction in veterinary medicine (11), caution will be needed for feline patients with a history of seizures. Recently, KET has received increasing attention in the management of RSE (2). However, our results emphasize that “Do not use ketamine as first line for treatment of status epilepticus (or seizure management)” and “use ketamine after confirmation of GABAergic/benzodiazepine-resistant (refractory) status epilepticus.” Whether KET is an anti-seizure or pro-seizure medication, the present results indicate that KET has a proconvulsant feature for interictal cats with TLE and/or high seizure frequency.

## Data availability statement

The raw data supporting the conclusions of this article will be made available by the authors, without undue reservation.

## Ethics statement

This study, including care and maintenance of the FSEC colony, healthy, and epileptic cats genetically unrelated to FSECs, was reviewed and approved by the Animal Care and Use Committee of Nippon Veterinary and Life Science University (accession Nos. 2020K-3, 2021K-2, 2022K-2; the principal investigator is DH).

## Author contributions

SM, RA, and DH designed this study. SM and RA conducted experiments, data acquisition, and analyses. SM wrote the draft of the manuscript and performed statistics. SM and YY performed data visualization. YH, YY, and DH performed editing of the draft. All authors contributed to the article and approved the submitted version.

## Funding

This study was partially supported by the Science Research Promotion Fund (grant numbers 2022-16 and 2023-18) from the Promotion and Mutual Aid Corporation for Private School of Japan (PMAC).

## Conflict of interest

The authors declare that the research was conducted in the absence of any commercial or financial relationships that could be construed as a potential conflict of interest.

## Publisher’s note

All claims expressed in this article are solely those of the authors and do not necessarily represent those of their affiliated organizations, or those of the publisher, the editors and the reviewers. Any product that may be evaluated in this article, or claim that may be made by its manufacturer, is not guaranteed or endorsed by the publisher.
