# Supplementary material for: Investigation of the effect and availability of ketamine on electroencephalography in cats with temporal lobe epilepsy
Source: Front Vet Sci. 2023 Jul 25;10:1236275. doi: 10.3389/fvets.2023.1236275 (PMC10407800; doi:10.3389/fvets.2023.1236275)
Supplement: Supplementary file 1 [file Table_1.pdf]

Supplementary Table 1.

Signalment and number of seizures per year for individual cats included in the study. These data refer to the condition at the time of experimental inclusion.

Cat (No)	Age (Months)	Sex	Body weight (kg)	Seizure frequency (/year)
TLE group				
1	142	Male	3.6	12
2	132	Male	4.1	19
3	131	Male	6.0	0
4	109	Female*	4.1	1
5	126	Male	3.1	0
6	139	Male	3.4	0
7	130	Female*	3.1	0
8	137	Female	2.5	0
9	100	Male	5.6	0
10	105	Female*	3.2	0
11	110	Male	5.3	0
12	97	Male	4.1	0
13	94	Female	3.6	0
14	151	Male	3.3	0
15	103	Male	5.0	0
16	157	Male	3.4	0
17	105	Female	2.8	0
18	69	Male	7.6	14
Control group				
19	107	Female*	3.6	—
20	107	Female*	5.4	—
21	94	Male	3.9	—
22	94	Male	3.1	—
23	94	Female	3.6	—
24	94	Male	6.4	—

Seizure frequency of TEL group was the seizure count of the last year before this study. All cats of TLE group had more than 2 seizures in their life, even if the value “0” was recorded.

*, Neutered
