# Supplementary material for: Investigation of the effect and availability of ketamine on electroencephalography in cats with temporal lobe epilepsy
Source: Front Vet Sci. 2023 Jul 25;10:1236275. doi: 10.3389/fvets.2023.1236275 (PMC10407800; doi:10.3389/fvets.2023.1236275)
Supplement: Supplementary file 2 [file Table_2.pdf]

Supplementary Table 2.

Results of relative power obtained from analysis of background activity under medetomidine alone and ketamine following medetomidine (medetomidine-ketamine) in the TLE group and control group.

TLE group		Medetomidine Median relative power (inter-quartile range)	Medetomidine-Ketamine Median relative power (inter-quartile range)	P value	Control group		Medetomidine Median relative power (inter-quartile range)	Medetomidine-Ketamine Median relative power (inter-quartile range)	P value
Delta (0.5–4.0 Hz)	F3	61.20 (50.37–66.87)	80.87 (67.42–86.67)	**	Delta (0.5–4.0 Hz)	F3	54.41 (47.58–61.74)	82.40 (69.28–87.32)	*
	Fz	61.63 (22.44–61.63)	83.58 (74.16–88.93)	**		Fz	54.20 (49.60–56.68)	88.86 (79.285–89.45)	*
	F4	57.67 (49.08–64.28)	75.98 (66.80–86.41)	*		F4	53.29 (50.16–62.10)	84.47 (78.36–90.43)	*
	C3	53.00 (44.74–63.22)	68.61 (54.46–77.92)	*		C3	46.24 (35.53–59.94)	86.25 (77.16–88.37)	*
	Cz	64.52 (56.53–71.48)	87.51 (70.08–92.76)	*		Cz	58.08 (46.79–64.96)	90.63 (87.40–92.32)	*
	C4	49.56 (41.54–66.63)	76.25 (51.58–83.59)	*		C4	47.28 (39.50–60.81)	79.00 (71.56–85.50)	*
	T3	63.00 (56.52–69.54)	86.08 (66.57–90.98)	*		T3	54.18 (52.44–62.40)	85.26 (81.34–89.42)	*
	T4	62.49 (52.09–65.51)	76.95 (64.60–92.52)	*		T4	49.05 (37.43–59.66)	90.19 (86.60–90.91)	*
	O1	55.14 (47.67–67.21)	81.89 (73.75–87.60)	**		O1	60.21 (46.77–66.04)	87.09 (84.21–91.92)	*
	Pz	65.12 (51.49–73.09)	81.06 (76.65–88.83)	**		Pz	58.27 (54.37–71.09)	90.38 (86.56–93.49)	*
	O2	55.22 (51.42–63.99)	82.43 (72.13–88.77)	**		O2	48.77 (45.78–55.49)	87.80 (79.65–89.35)	*
Theta (4.1–8.0 Hz)	F3	16.80 (13.84–23.81)	7.42 (5.37–11.16)	**	Theta (4.1–8.0 Hz)	F3	18.45 (16.07–20.99)	10.20 (7.56–15.95)	NS
	Fz	20.04 (16.70–22.07)	8.61 (5.90–12.82)	**		Fz	22.93 (19.59–27.59)	7.40 (4.90–12.58)	*
	F4	18.88 (13.45–20.87)	9.89 (6.65–12.21)	*		F4	17.29 (14.44–21.51)	7.33 (4.86–9.94)	*
	C3	18.61 (14.95–20.82)	14.28 (10.47–20.54)	NS		C3	17.56 (14.81–22.79)	6.38 (5.71–7.87)	*
	Cz	15.71 (13.45–18.50)	5.40 (4.40–14.32)	*		Cz	19.59 (17.46–21.64)	6.33 (5.22–7.75)	*

	C4	18.93 (14.47–20.65)	11.40 (6.32–17.17)	*		C4	23.72 (17.97–28.23)	8.65 (7.04–12.62)	*
	T3	17.08 (13.08–20.44)	7.50 (5.18–12.20)	*		T3	20.45 (17.40–23.32)	8.30 (7.44–9.75)	*
	T4	16.69 (15.44–18.82)	8.92 (5.14–13.97)	*		T4	20.07 (19.82–21.51)	5.97 (5.62–6.47)	*
	O1	20.26 (16.77–21.33)	9.11 (6.61–12.19)	*		O1	20.98 (17.52–23.97)	7.20 (5.08–10.07)	*
	Pz	16.58 (12.83–20.30)	9.41 (6.21–15.11)	*		Pz	20.05 (19.27–20.88)	5.60 (4.23–10.42)	*
	O2	19.10 (14.85–22.56)	8.29 (6.14–13.35)	**		O2	21.45 (19.42–25.61)	7.11 (5.26–10.95)	*
Alpha (8.1–13.0 Hz)	F3	8.47 (5.78–11.71)	3.27 (1.80–4.62)	**	Alpha (8.1–13.0 Hz)	F3	11.52 (11.24–13.79)	3.11 (1.86–5.90)	*
	Fz	9.01 (7.43–12.73)	2.94 (2.43–5.48)	**		Fz	12.72 (8.97–15.77)	2.02 (1.72–3.02)	*
	F4	9.34 (6.09–11.24)	3.91 (2.46–4.84)	**		F4	15.77 (14.94–16.07)	2.97 (1.90–3.78)	*
	C3	10.80 (9.90–14.40)	6.60 (3.00–8.80)	**		C3	20.40 (12.46–28.03)	2.60 (2.02–3.82)	*
	Cz	7.74 (6.15–10.50)	2.35 (1.36–4.75)	**		Cz	17.67 (11.77–24.08)	1.88 (1.40–2.00)	*
	C4	9.56 (6.75–14.06)	3.94 (2.23–8.03)	*		C4	13.82 (13.33–19.88)	3.59 (1.92–5.60)	*
	T3	6.99 (6.20–10.27)	3.17 (1.76–4.83)	*		T3	14.91 (10.15–19.23)	2.54 (2.22–3.80)	*
	T4	9.84 (6.83–12.53)	3.46 (1.61–6.10)	**		T4	20.43 (12.20–27.99)	2.02 (1.82–2.30)	*
	O1	10.38 (7.46–12.05)	4.74 (2.01–5.17)	**		O1	12.25 (9.03–16.99)	2.57 (1.32–3.25)	*
	Pz	8.69 (6.10–10.28)	2.90 (1.97–4.40)	**		Pz	11.00 (6.95–14.33)	1.89 (1.18–2.11)	*
	O2	8.04 (5.89–12.94)	3.72 (2.19–5.24)	**		O2	15.33 (12.49–17.02)	2.71 (1.58–3.04)	*
Beta (13.1–30.0 Hz)	F3	8.66 (5.75–20.28)	4.89 (3.92–9.58)	NS	Beta (13.1–30.0 Hz)	F3	9.98 (6.73–13.40)	5.67 (4.03–8.87)	NS
	Fz	6.25 (3.78–10.52)	3.48 (2.20–6.61)	NS		Fz	5.44 (4.57–6.86)	2.85 (1.89–4.62)	*
	F4	10.03 (6.17–18.78)	4.98 (3.67–8.28)	NS		F4	6.77 (5.61–11.66)	5.24 (2.34–8.42)	*
	C3	15.00 (9.60–25.80)	6.00 (3.30–15.10)	*		C3	9.50 (8.27–10.66)	3.90 (3.40–7.82)	*

	Cz	6.70 (4.38–15.51)	2.29 (1.83–6.04)	*		Cz	4.72 (4.37–8.64)	2.16 (1.04–3.31)	*
	C4	13.45 (7.29–25.44)	5.97 (3.46–16.09)	NS		C4	9.00 (6.82–14.92)	9.10 (4.42–11.03)	NS
	T3	10.15 (7.02–18.46)	4.50 (2.08–8.93)	*		T3	8.40 (7.32–9.38)	3.51 (1.88–6.02)	*
	T4	11.96 (6.84–16.64)	2.97 (2.20–10.48)	*		T4	8.13 (7.46–10.82)	2.02 (1.60–4.97)	*
	O1	8.11 (6.00–19.35)	3.41 (2.45–6.89)	*		O1	7.58 (5.34–9.30)	2.17 (1.66–3.48)	*
	Pz	4.93 (4.06–11.30)	3.36 (1.8–4.51)	*		Pz	4.84 (3.14–6.08)	1.47 (0.78–2.26)	*
	O2	9.51 (5.39–13.96)	4.45 (2.92–7.67)	*		O2	8.98 (8.08–9.79)	3.37 (2.52–4.03)	*

Statistical analysis was performed with Wilcoxon signed rank test. Statistical analyses were performed using R version 4.1.0 (The R Foundation for Statistical Computing, Vienna, Austria). The significant difference in statistical analysis was defined as $p < 0.05$.

NS, non-significant. Significant: *, $P < 0.05$, **, $P < 0.001$
